# CO-dependent hydrogen production by the facultative anaerobe *Parageobacillus thermoglucosidasius*

**DOI:** 10.1186/s12934-018-0954-3

**Published:** 2018-07-09

**Authors:** Teresa Mohr, Habibu Aliyu, Raphael Küchlin, Shamara Polliack, Michaela Zwick, Anke Neumann, Don Cowan, Pieter de Maayer

**Affiliations:** 1 Section II: Technical Biology, Institute of Process Engineering in Life Science, Karlsruhe Institute of Technology, 76131 Karlsruhe, Germany; 2 Centre for Microbial Ecology and Genomics, University of Pretoria, Hatfield 0028 Pretoria, South Africa; 3 School of Molecular & Cell Biology, Faculty of Science, University of the Witwatersrand, WITS 2050 Johannesburg, South Africa; 4 Section II: Technical Biology, Institute of Process Engineering in Life Science, Karlsruhe Institut für Technologie (KIT), Kaiserstrasse 12, 76131 Karlsruhe, Germany

**Keywords:** Biohydrogen production, *Parageobacillus thermoglucosidasius*, Carbon monoxide dehydrogenase, Hydrogenase, Water-gas shift reaction

## Abstract

**Background:**

The overreliance on dwindling fossil fuel reserves and the negative climatic effects of using such fuels are driving the development of new clean energy sources. One such alternative source is hydrogen (H_2_), which can be generated from renewable sources. *Parageobacillus thermoglucosidasius* is a facultative anaerobic thermophilic bacterium which is frequently isolated from high temperature environments including hot springs and compost.

**Results:**

Comparative genomics performed in the present study showed that *P. thermoglucosidasius* encodes two evolutionary distinct H_2_-uptake [Ni-Fe]-hydrogenases and one H_2_-evolving hydrogenases. In addition, genes encoding an anaerobic CO dehydrogenase (CODH) are co-localized with genes encoding a putative H_2_-evolving hydrogenase. The co-localized of CODH and uptake hydrogenase form an enzyme complex that might potentially be involved in catalyzing the water-gas shift reaction (CO + H_2_O → CO_2_ + H_2_) in *P. thermoglucosidasius*. Cultivation of *P. thermoglucosidasius* DSM 2542^T^ with an initial gas atmosphere of 50% CO and 50% air showed it to be capable of growth at elevated CO concentrations (50%). Furthermore, GC analyses showed that it was capable of producing hydrogen at an equimolar conversion with a final yield of 1.08 H_2_/CO.

**Conclusions:**

This study highlights the potential of the facultative anaerobic *P. thermoglucosidasius* DSM 2542^T^ for developing new strategies for the biohydrogen production.

**Electronic supplementary material:**

The online version of this article (10.1186/s12934-018-0954-3) contains supplementary material, which is available to authorized users.

## Background

In the next 30 years, the global energy demand will expand by ca. 30% and the vast majority (ca. 85%) of the energy resources required to offset the rising demand will come from non-renewable sources such as natural gas and crude oil [1]. This will result in increased pressure on the dwindling fossil fuel reserves and in greater emission of greenhouse gases into the Earth’s atmosphere. There is thus an urgent need for further development and implementation of clean and renewable alternative energy sources [2, 3].

Hydrogen (H_2_) has recently become prominent as a very attractive clean and sustainable energy source, especially when generated via ‘eco-friendly’ strategies. In comparison to other fuels, H_2_ has the highest energy content (141.9 MJ/kg higher heating value) [2]. Additionally, its complete combustion with pure oxygen produces only water (2 H_2_ + O_2_ → 2 H_2_O) as a by-product. The majority of current industrial H_2_ production strategies, such as coal gasification, steam reformation and partial oxidation of oil, are unsustainable, harmful to the environment, energy intensive and expensive [4, 5]. As such, over the past few years, the production of H_2_ via microbial catalysis has drawn increasing interest. Several different strategies to produce biohydrogen, such as photofermentation of organic substances by photosynthetic bacteria, bio-photolysis of water by algae and dark fermentation of organic substances by anaerobic microorganism, have been explored [6]. These strategies have the advantage of lower energy expenditure, lower cost and higher yields than the industrial methods [7]. Another advantage is the potential to use cheap feedstocks, such as lignocellulosic waste biomass, which can be converted into a gas mixture termed ‘synthesis gas’. This gas consists primarily of carbon monoxide (CO), carbon dioxide (CO_2_) and H_2_ [8]. In a further step, the CO can react with water to generate H_2_ via a biologically- or chemically-mediated water-gas shift (WGS) reaction: CO + H_2_O → CO_2_ + H_2_. During the biologically mediated reaction, a carbon monoxide dehydrogenase (CODH) oxidizes CO and electrons are released. Subsequently, a coupled hydrogenase reduces the released electrons to molecular hydrogen [9]. Several mesophilic, anaerobic prokaryotic taxa, including *Rhodospirillum rubrum* and *Rhodopseudomonas palustris*, are known for the ability to perform the WGS reaction [10].

It has been observed that higher yields of H_2_ can be obtained in higher temperature fermentations [11]. Thus, there has been increasing interest in the use of thermophilic anaerobic bacteria, such as *Carboxydothermus hydrogenoformans* and *Thermosinus carboxydivorans* [12, 13], as well thermophilic archaea (e.g. *Thermococcus onnurineus*) [7].

An industrial process utilizing CO-oxidizing bacteria for biohydrogen production has not yet been realized, although many CO-using hydrogenogenic species have been isolated. This may largely be attributed to the sensitivity of both the hydrogenase and CODH enzymes to oxygen [6, 14]. For example, the hydrogenase of *Thermotoga maritima* lost 80% of its activity after flushing with air for 10 s [15]. Removal of O_2_ from industrial waste gases or from bioreactors is prohibitively expensive, making the use of strictly anaerobic CO-oxidizing hydrogenogens unfeasible [16]. Here, we have analysed the hydrogenogenic capacity of the facultative anaerobe *Parageobacillus thermoglucosidasius*. Comparative genomics revealed the presence of three distinct hydrogenases, two uptake hydrogenases as well as one H_2_-evolving hydrogenase, which is linked to an anaerobic CODH. Evolutionary analysis showed that this combination of hydrogenases is unique to *P. thermoglucosidasius* and suggests that H_2_ plays a pivotal in the bioenergetics of this organism. Furthermore, fermentations and downstream GC analysis showed that *this* facultative anaerobe is capable of utilizing CO in the WGS reaction to generate an equimolar amount of H_2_ once most of the oxygen in the medium has been exhausted.

## Methods

### Microorganisms

The production of H_2_ by *P. thermoglucosidasius* when grown in the presence of CO was tested using *P. thermoglucosidasius* DSM 2542^T^. Two related strains, *Geobacillus thermodenitrificans* DSM 465^T^ and *P. toebii* DSM 14590^T^, which lack orthologues of the three hydrogenase loci as well as the CODH locus, were included as controls. All strains were obtained from the DSMZ (Deutsche Sammlung von Mikroorganismen und Zellkulturen GmbH, Braunschweig, Germany).

### Culture conditions and media

Pre-cultures and cultures were grown aerobically in mLB (modified Luria–Bertani) medium containing tryptone (1% w/v), yeast extract (0.5% w/v), NaCl (0.5% w/v), 1.25 ml/l NaOH (10% w/v), and 1 ml/l of each of the filter-sterilized stock solutions: 1.05 M nitrilotriacetic acid, 0.59 M MgSO_4_·7H_2_O, 0.91 M CaCl_2_·2H_2_O and 0.04 M FeSO_4_·7H_2_O. The first pre-culture was inoculated from glycerol stock (20 µl in 20 ml mLB) and cultivated for 24 h at 60 °C and rotation at 120 rpm in an Infors Thermotron (Infors AG, Bottmingen, Switzerland). A second pre-culture was inoculated from the first one to an OD_600_ of 0.1 and incubated as above for 12 h. For the experiments, 250 ml serum bottles were prepared with 50 ml mLB and a gas phase of 50% CO and 50% atmospheric air at 1 bar atmospheric pressure, which were inoculated with 1 ml from the second pre-culture. The experiments were conducted in quadruplicate for a total duration of 84 h.

### Analytical methods

Samples were taken at different time points during the experimental procedure. Before and after the sampling the pressure was measured using a manometer (GDH 14 AN, Greisinger electronic, Regenstauf, Germany). To monitor the growth of the cultures, 1 ml of the culture was aspirated through the stopper and absorbance was measured at OD_600_ using an Ultrospec 1100 pro spectrophotometer (Amersham Biosciences, USA). The determination of the gas compositions at different time points was conducted using a 3000 Micro GC gas analyzer (Inficon, Bad Ragaz, Switzerland) with the columns Molsieve and PLOT Q. A total of 3 ml was sampled from the head space and injected into the GC. A constant temperature of 80 °C was maintained during the total analysis time of 180 s. The gas compositions at the different sampling points were calculated using the formulas in Additional file 1.

### Comparative genomic analyses

The large hydrogenase subunits were identified from the annotated genome of *P. thermoglucosidasius* DSM 2542^T^ (CP012712.1) by comparison against the Hydrogenase DataBase (HyDB) [17]. The full hydrogenase loci were identified by searching the genome up- and downstream of the large subunit gene, extracted and mapped against the genome using the CGView server [18]. The proteins encoded on the genome were compared by BlastP against the NCBI non-redundant (nr) protein database to identify orthologous loci. Full loci were extracted from the comparator genomes and all loci were structurally annotated using Genemark.hmm prokaryotic v.2 [19]. The resultant protein datasets were compared by local BlastP with Bioedit v 7.2.5 [20] to identify orthologues, where orthology was assumed for those proteins sharing > 30% amino acid identity over 70% sequence coverage.

A maximum likelihood (ML) phylogeny was constructed based on the amino acid sequences of three commonly used housekeeping markers: translation initiation factor IF-2 (InfB), DNA recombination and repair protein RecN RNA polymerase subunit B (RpoB). The proteins were individually aligned using M-Coffee [21], the alignments concatenated and poorly aligned regions were trimmed using Gblocks [22]. Finally, the trimmed alignment was used to generate a ML phylogeny using PhyML-SMS, using the optimal amino acid substitution model as predicted by the Smart Model Selection tool [23, 24]. Similarly, ML phylogenies were constructed on the basis of the concatenated orthologous proteins encoded on the Pha, Phb, Phc and CODH loci.

## Results

### The genome of *P. thermoglucosidasius* encodes three distinct hydrogenases

**Fig. 1:**
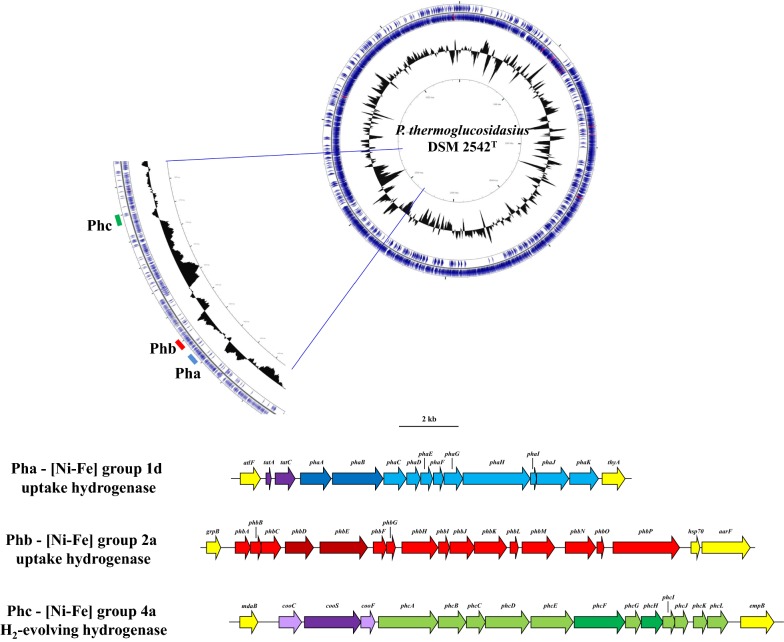
Schematic diagram of the [Ni-Fe] hydrogenase loci and their localization on the chromosome of *P. thermoglucosidasius* DSM 2542^T^

Analysis of the complete, annotated genome sequence of *P. thermoglucosidasius* DSM 2542^T^ showed the presence of three putative [Ni-Fe]-hydrogenase loci on the chromosome. Two of these hydrogenases are encoded on the forward strand, while the third is located on the reverse strand (Fig. 1). Given the convoluted nomenclature of hydrogenase genes, we have termed these loci as ***P****arageobacillus* **h**ydrogenase **a**, **b** and **c**, in accordance with their chromosomal locations), to distinguish between them.

The Pha locus (chromosomal position 2,456,963–2,469,832; 12.9 kb in size) comprises eleven protein coding sequences (NCBI Accession ALF10692-10702; PhaA-PhaK) (Fig. 1; Additional file 2). Comparison of the amino acid sequence of the predicted catalytic subunit (ALF10727—PhaB) against HydDB [17] classifies the hydrogenase produced by this locus as a [Ni-Fe] group 1d uptake hydrogenase (E-value = 0.0). This unidirectional, membrane-bound, O_2_-tolerant hydrogenase is present in a broad range of obligately aerobic and facultatively anaerobic soil-borne, aquatic and host-associated taxa such as *Ralstonia eutropha*, *Escherichia coli* and *Wolinella succinogenes* [25, 26]. The H_2_ molecules consumed by group 1d hydrogenases are coupled to aerobic respiration (O_2_ as electron acceptor) or to respiratory reduction of various anaerobic electron acceptors including NO^3−^, SO_4_^2−^, fumarate and CO_2_. The *P. thermoglucosidasius* DSM 2542^T^ hydrogenase locus incorporates genes coding for both small (PhaA; ALF10692; 324 aa) and large (PhaB; ALF10693; 573 aa) catalytic hydrogenase subunits. The strain also encodes seven additional proteins involved in hydrogenase formation, maturation and incorporation of the Ni-Fe metallocenter, including a third hydrogenase subunit (PhaC) which is predicted to serve as cytochrome *b* orthologue and links the hydrogenase to the quinone pools of the respiratory chains (Fig. 1; Additional file 2) [26]. The *pha* genes are flanked at the 5′ end by two genes coding for orthologues of the Twin-arginine translocation (Tat) pathway proteins TatA and TatC (Fig. 1; Additional file 2). These have been shown to form part of the membrane targeting and translocation (Mtt) pathway which targets the fully folded hydrogenase heterodimer to the membrane [27].

The Phb locus (chromosomal position 2,488,614–2,503,714; 15.1 kb in size), located ~ 19 kb downstream of the Pha locus, comprises 16 protein coding sequences (NCBI Accession ALF10723-738; PhbA-PhbP) (Fig. 1; Additional file 2). The predicted catalytic subunit (ALF10727—PhbE) compared against HydDB classifies the product of this locus as a [Ni-Fe] group 2a uptake hydrogenase (E-value = 0.0) [17]. Members of this group of uptake hydrogenases are widespread among aerobic soil bacteria and Cyanobacteria and play a role in recycling H_2_ produced by nitrogenase activity and fermentative pathways [28, 29]. The recycled H_2_ is used in hydrogenotrophic respiration with O_2_ serving as terminal electron acceptor, and thus group 2a hydrogenases are often O_2_-tolerant [26]. This locus encodes both large (PhbE; ALF10727; 544 aa) and small (PhbD; ALF10726; 317) [Ni-Fe] hydrogenase subunits and eight additional proteins with predicted roles in hydrogenase formation, maturation and incorporation of the Ni-Fe metal center in the large subunit (Fig. 1; Additional file 2) [26]. Furthermore, this locus encodes six proteins whose role in hydrogenase biosynthesis and functioning remains unclear. These include a tetratricopeptide-repeat (PhbH) and NHL repeat (PhbK) containing protein, which also occur in the [Ni-Fe] group 2a hydrogenase loci of *Nostoc punctiforme* ATCC 29133 and *Nostoc* sp. PCC 7120, where they are co-transcribed with the hydrogenase genes and have been suggested to play a role in protein–protein interactions and Fe–S cluster biogenesis (PhbJ) which may mediate electron transport to redox partners in downstream reactions [30].

The Phc locus (chromosomal position 2,729,489–2,741,372), ~ 226 kb downstream of the *Phb* locus is 11.9 kb in size and encodes 12 distinct proteins (PhcA-PhcL) (Fig. 1; Additional file 2). These include a small (PhcE; ALF10919; 247 aa) and large (PhcG; ALF19021; 574 aa) [Ni-Fe]-hydrogenase catalytic subunit and ten additional proteins involved in hydrogenase formation and maturation (Fig. 1; Additional file 2). The HydDB classifies the Phc hydrogenase as a [Ni-Fe] group 4a hydrogenase or formate hydrogenlyase complex I (FHL-1) [17]. Members of this group are oxygen-sensitive, membrane-bound and are largely restricted to the facultatively fermentative Proteobacteria, particularly enterobacteria associated with animal intestinal tracts [25, 26]. FHL-1 couples the reduction of protons from water to the anaerobic oxidation of formate to form CO_2_ and H_2_ [26, 31].

BlastP and tBlastN analyses of the protein sequences encoded in the *P. thermoglucosidasius* DSM 2542^T^ hydrogenase loci showed that the Pha, Phb and Phc loci are universally present in eight other *P. thermoglucosidasius* strains for which genomes are available (Additional file 2: Table S1). These loci are highly syntenous and the encoded proteins share average amino acid identities of 99.73% ([Ni-Fe]-group 1d hydrogenase Pha—13 proteins), 99.61% ([Ni-Fe]-group 2a hydrogenase Phb—16 proteins) and 99.22% ([Ni-Fe]-group 4a hydrogenase Phc—12 proteins) with those of DSM 2542^T^, respectively. Pairwise BlastP analyses showed limited orthology between the two uptake hydrogenase loci, with 36.29% average amino acid identity in nine proteins encoded on the two loci. The group 1d (Pha) and group 2a (Phb) uptake hydrogenase loci share 33.40 and 62.32% average amino acid identity for three proteins with the H_2_ evolving hydrogenase (Phc) locus. The higher level of orthology for Phb and Phc loci proteins can be correlated with the HypA-like (PhbB and PhcK) and the HypB-like (PhbC and PhcL) proteins, which share 75.22 and 86.08% amino acid identity, respectively, and are predicted to play a role in the incorporation of nickel into the hydrogenase enzyme [32]. Limited orthology is observed between the hydrogenase catalytic subunits or other hydrogenase formation and maturation proteins, suggesting distinct evolutionary histories for the two uptake and one H_2_-evolving hydrogenases in *P. thermoglucosidasius*.

### *P. thermoglucosidasius* contains a unique profile of hydrogenases with distinct evolutionary histories

**Fig. 2:**
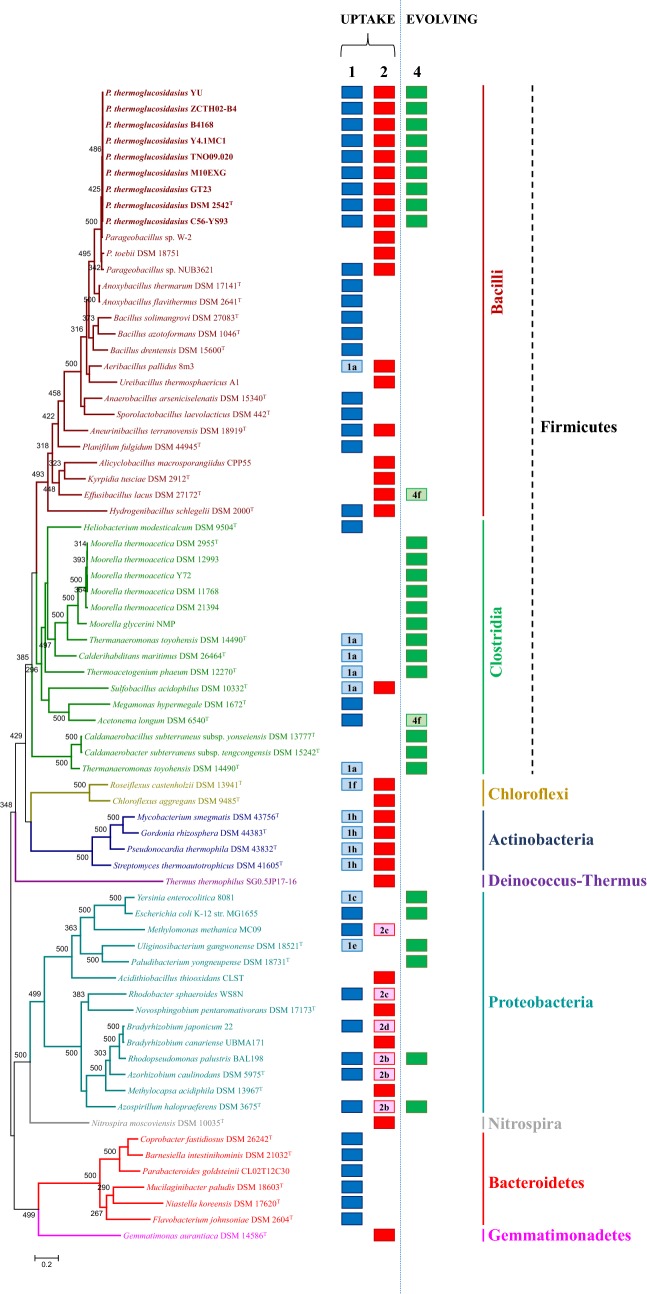
Prevalence of [Ni-Fe] hydrogenases orthologous to those in *P. thermoglucosidasius* among other bacterial taxa. The ML phylogeny was constructed on the basis of the trimmed alignment (1597 amino acids in length) of the concatenated InfB, RecN and RpoB amino acid sequences. Different taxa and branch colours indicate the different phyla/classes. Values on the branches indicate bootstrap values (n = 500 replicates) and the tree was rooted on the midpoint. The presence of [Ni-Fe] group 1d, group 2a and group 4a hydrogenases is represented by dark blue, red and green blocks, respectively. Where [Ni-Fe] hydrogenases belonging to the same groups but not the same subtype as those in *P. thermoglucosidasius* are present they are indicated by light blue ([Ni-Fe] group 1 hydrogenases), pink ([Ni-Fe] group 2 hydrogenases) and light green ([Ni-Fe] group 4 hydrogenases) blocks, respectively

The proteins encoded by the Pha, Phb and Phc loci were used in BlastP comparisons against the NCBI non-redundant (nr) protein database and HydDB (catalytic subunits) to identify orthologous loci in other bacterial taxa. This revealed that, aside from the α-proteobacteria *Azospirillum halopraeferens* DSM 3675^T^ and *Rhodopseudomonas palustris* BAL398, the combination of [Ni-Fe] group 1- 2- 4 hydrogenases appears to be unique to *P. thermoglucosidasius* (Fig. 2). In these two proteobacterial taxa the group 2a uptake hydrogenase is, however, replaced by a group 2b uptake hydrogenase. Group 2b uptake hydrogenases do not have a direct role in energy transduction but are flanked by a PAS domain protein which accepts the hydrogenase-liberated electrons, modulating the activity of a two-component regulator that upregulates the expression of other uptake hydrogenases, thereby serving as H_2_-sensing system [33, 34].

**Fig. 3:**
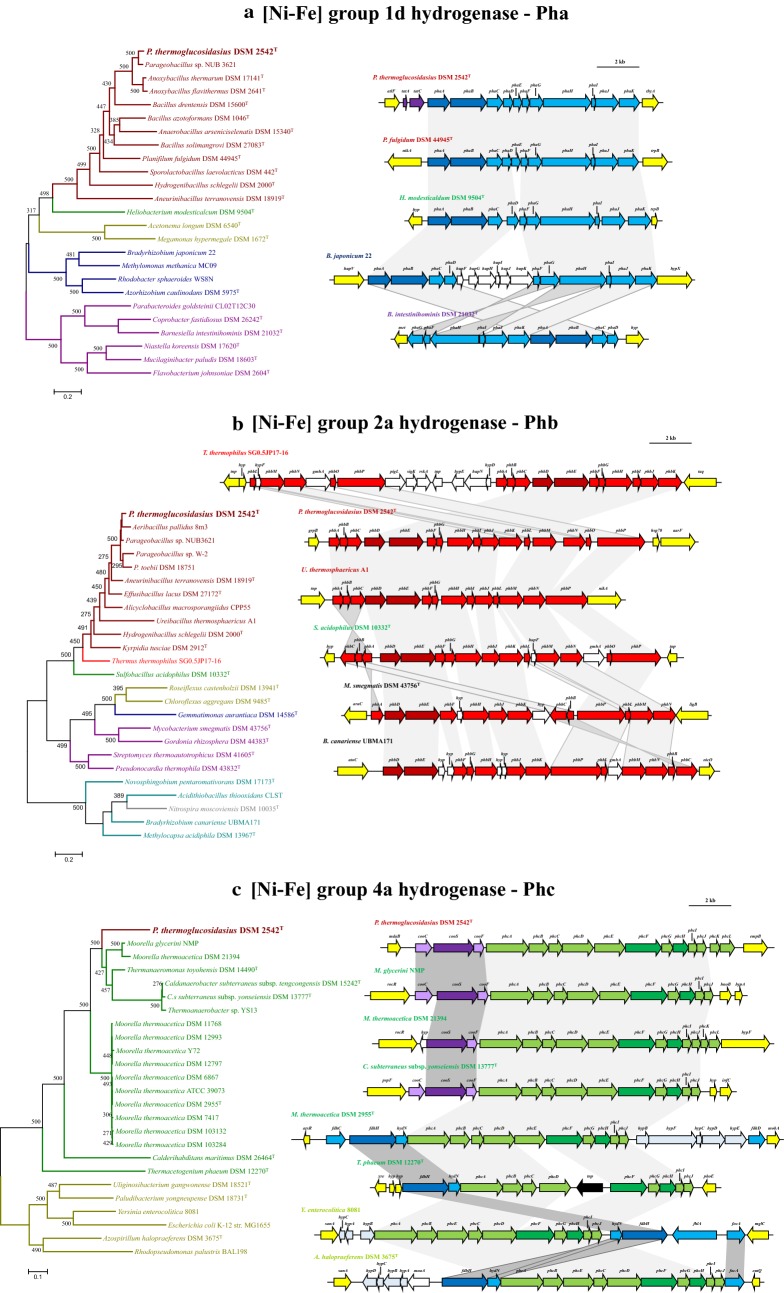
Prevalence and synteny of the *P. thermoglucosidasius*-like [Ni-Fe] hydrogenases. **a** [Ni-Fe] group 1d orthologues. The ML phylogeny was determined on the basis of the trimmed alignment of nine Pha locus proteins (PhaABCDGHIJK—2206 amino acids in length). Hydrogenase genes are coloured in light blue (dark blue for large and small catalytic subunits), *tatAE* genes in purple and flanking genes in yellow in the synteny diagrams. **b** [Ni-Fe] group 2a orthologues. The ML phylogeny was determined on the basis of the trimmed alignment of 10 Phb locus proteins (PhbBCDEFHJLMN—2348 amino acids in length). Hydrogenase genes are coloured in red (dark red for large and small catalytic subunits), genes of no known function in biosynthesis and functioning of the hydrogenase in white and flanking genes in yellow in the synteny diagrams. **c** [Ni-Fe] group 4a orthologues. The ML phylogeny was determined on the basis of the trimmed alignment of nine Phc locus proteins (PhcABCDFGHIJ—2744 amino acids in length). Hydrogenase genes are coloured in light green (dark green for large and small catalytic subunits), anaerobic CODH genes in purple, formate dehydrogenase-related genes in blue and flanking genes in yellow in the synteny diagrams. Values on all trees reflect bootstrap analyses (n = 500 replicates) and all trees were rooted on the midpoint

The Pha uptake hydrogenase locus is relatively well conserved among members of the Firmicutes, including a number of taxa belonging to the Classes Bacilli, Clostridia and Negativicutes, as well as the phyla Proteobacteria and Bacteroidetes (Fig. 2; Additional file 3). However, the more distantly related taxa retain little synteny with the Pha locus in *P. thermoglucosidasius* (Fig. 3a). Orthologues of the Pha are present in one other *Parageobacillus* spp., namely genomosp. NUB3621, with an average amino acid identity of 92.37% (13 proteins) with the DSM 2542^T^ Pha proteins. A phylogeny on the basis of nine conserved Pha proteins (PhaABCDGHIJK) showed a similar branching pattern (Fig. 3a) as observed for the phylogeny housekeeping protein (InfB-RecN-RpoB) phylogeny, suggesting that this is an ancestral locus that has been vertically maintained. This is supported by the low level of discrepancy in G+C content for the *P. thermoglucosidasius* strains, which are on average 0.87% above the genomic G+C content. Larger discrepancies are, however, evident among the Bacteroidetes, where G+C contents for the locus are on average 4.43% above that of the genome, and the absence of Pha loci in other *Parageobacillus* spp. including *P. toebii* (five genomes available) and *P. caldoxylosilyticus* (four genomes available) and *Geobacillus* spp. suggest a more complex evolutionary history for the group 1d hydrogenase.

Orthologous [Ni-Fe] group 2a uptake hydrogenase (Phb) loci are also common among the Firmicutes, but show a more restricted distribution within the family *Bacillaceae*, with only *Aeribacillus pallidus* 8m3 and *Hydrogenibacillus schlegelii* DSM 2000^T^ containing orthologues outside the genus *Parageobacillus*. Highly conserved and syntenous loci are, however, present in three non-*thermoglucosidasius* strains: *Parageobacillus* sp. NUB3621, *Parageobacillus* sp. W-2 and *P. toebii* DSM 18751 (Fig. 3b; Additional file 3). Orthologous loci are present across a much wider range of phyla than the Pha locus, including members of the Chloroflexi, Gemmatimonadetes, Actinobacteria, Proteobacteria, Nitrospirae and Deinococcus-Thermus (Fig. 2). The latter is of interest as *Thermus thermophilus* SG0.5JP17-16 clusters with the Firmicutes in a phylogeny of ten conserved proteins (PhbBCDEFHJLMN—72.76% average amino acid identity with *P. thermoglucosidasius* DSM 2542^T^) (Fig. 3b), but is phylogenetically disparate from the Firmicutes. The *T. thermophilus* locus is present on the plasmid pTHTHE1601 (NC_017273) suggesting that this locus forms part of the mobilome. Furthermore, the G+C content of the Phb locus differs by an average of 4.55% from the average genomic G+C among the eight compared *P. thermoglucosidasius* strains, suggesting recent horizontal acquisition of this locus.

The [Ni-Fe] group 4a H_2_-evolving hydrogenase (Phc) locus shows the most restricted distribution of the three loci among the Firmicutes, with orthologous loci only present in the eight compared *P. thermoglucosidasius* strains and members of the clostridial family *Thermoanaerobacteraceae* (Fig. 2). Further, Phc-like loci appear to be restricted to members of the Proteobacteria. High levels of synteny and sequence conservation can be observed among the Phc loci in both phyla, with the exception of the PhcK and PhcL proteins, which are only present in the *P. thermoglucosidasius* and *Moorella thermoacetica* DSM 21394 Phc loci (Fig. 3c). BlastP analyses indicate that PhcK and PhcL show highest orthology with PhbB and PhbC in the Phb locus and may have been derived through gene duplication events.

It is notable that the *P. thermoglucosidasius* Phc locus clusters with a subset of the *Thermoanaerobacteraceae* in the concatenated Phc protein phylogeny, including *Moorella glycerini* NMP, *M. thermoacetica* DSM 21394, *Thermoanaeromonas toyohensis* DSM 14490^T^, *Caldanaerobacter subterraneus* subsp. *tencogensis* DSM 15242^T^ and subsp. *yonseiensis* DSM 13777^T^ and *Thermoanaerobacter* sp. YS13 (Fig. 3c). These differ from the remaining *Thermoanaerobacteraceae* taxa and the proteobacterial orthologous loci in that they are flanked by three genes, *cooCSF,* coding for an anaerobic carbon monoxide (CO) dehydrogenase, rather than genes coding for a formate dehydrogenase (FdhH) as is typical for the [Ni-Fe] group 4a hydrogenases [25]. These are generally accompanied by flanking genes coding for the formate dehydrogenase accessory sulfurtransferase protein FdhD, electron transporter HydN, transcriptional activator FhlA and formate transporters FdhC and FocA, which together with FdhH drive the anaerobic oxidation of formate (Fig. 3c) [26, 35–37].

BlastP analysis with the FdhH protein of *M. thermoacetica* DSM 2955^T^ (AKX95035) shows that an orthologue is present in *P. thermoglucosidasius* DSM 2542^T^ (ALF09582). The latter protein, however, shares limited orthology (39% amino acid identity; Bitscore: 497; E-value: 6e−614) with its *M. thermoacetica* counterpart and is furthermore localised ~ 1.5 Mb upstream of the Phc locus, suggesting the *P. thermoglucosidasius* FdhH protein does not function together with the [Ni-Fe] group 4a hydrogenase. Instead, the *P. thermoglucosidasius* Phc hydrogenase may form a novel complex with the adjacent anaerobic CODH locus.

### The *P. thermoglucosidasius* [Ni-Fe] group 4a hydrogenase forms a novel complex with the anaerobic (Coo) CO dehydrogenase, with a distinct evolutionary history

**Fig. 4:**
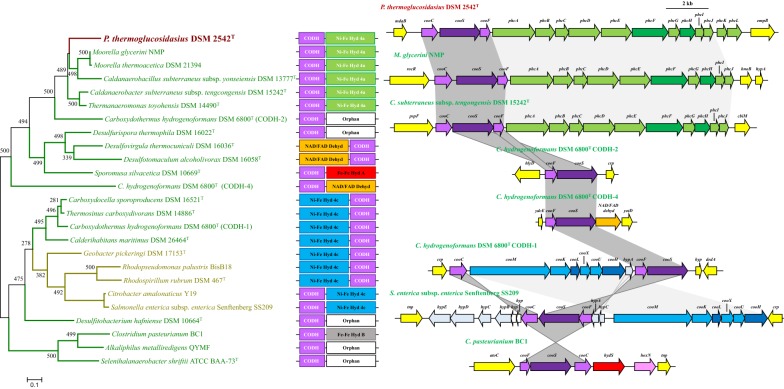
Prevalence and synteny of the *P. thermoglucosidasius*-like CODH loci. A phylogeny was constructed on the basis of the concatenated alignments of two proteins (CooFS—692 amino acids in length). Boostrap analysis (n = 500 replicates) was performed and the tree was rooted on the mid-point. In the synteny diagrams the CODH genes are coloured in purple (dark purple for the catalytic subunit gene *cooS*), the [Ni-Fe] group 4c hydrogenase genes in blue (dark blue for catalytic subunits), the [Ni-Fe] group 4a hydrogenase genes in green (dark green for catalytic subunits), NAD/FAD oxidoreductase gene in orange, [Fe-Fe] hydrogenase group A genes in red, [Fe-Fe] hydrogenase group B genes in purple and flanking genes in yellow

The three genes located just upstream of the Phc hydrogenase locus, *cooC*, *cooS* and *cooF* code for a CO dehydrogenase maturation factor (Figs. 3c, 4), a CO dehydrogenase catalytic subunit and CO dehydrogenase Fe–S protein, respectively. Together these proteins catalyse the oxidation of CO to generate CO_2_ (CO + H_2_O → CO_2_ + 2 H^+^ + 2ē). The electrons are then used in reduction reactions, including sulphate reduction, heavy metal reduction, acetogenesis, methanogenesis and hydrogenogenesis [38, 39].

The CODH locus is also co-localised with the Phc hydrogenase locus and highly conserved among the eight other *P. thermoglucosidasius* genomes (99.36% average amino acid identity with CooCSF in *P. thermoglucosidasius* DSM 2542^T^), while no CODH orthologues are encoded on the genomes of any other *Parageobacillus* or *Geobacillus* spp. A phylogeny on the basis of the conserved CooS and CooF proteins (Fig. 4) showed that, as with the Phc locus phylogeny (Fig. 3c), those taxa where *cooCFS* flanks the Phc hydrogenase locus cluster together and show extensive synteny in both the *coo* and *phc* gene clusters. This would suggest the co-evolution of the anaerobic CODH and Phc [Ni-Fe] group 4a hydrogenase loci. However, differences in the G+C contents could be observed among the *P. thermoglucosidasius coo* (average G+C content 46.97%) and *phc* (average G+C content 49.02%) loci. This is even more pronounced among the *Thermoanaerobacteraceae* with this CODH-Phc arrangement, where the G+C contents of the two loci differs by an average of 6.62% and is particularly evident in *C. subterraneus* subsp. *tencongensis* where the G+C contents of the *coo* and *phc* loci differ by 11.77%, suggesting independent evolution of these two loci. This is further support by the phylogeny (Fig. 4), where the CODH-Phc loci cluster with CODHs which appear on their own and those flanked by an NAD/FAD oxidoreductase are thought to play a role in oxidative stress response [40]. The Energy Conserving Hydrogenase (ECH–[Ni-Fe] group 4c hydrogenase)-CODH complex, which has been shown to couple CO oxidation to proton reduction to H_2_ in *C. hydrogenoformans* and *Rhodospirillum rubrum*, clusters more distantly from the CODH-[Ni-Fe] group 4a complex [41, 42]. Overall, the results suggest that the CODH and [Ni-Fe] group 4a hydrogenase have evolved independently, but may form a complex linking CO oxidation to reduction of protons to produce CO_2_ and H_2_.

### The CODH-[Ni-Fe] group 4a hydrogenase complex effectively couples CO oxidation to hydrogenogenesis

**Fig. 5:**
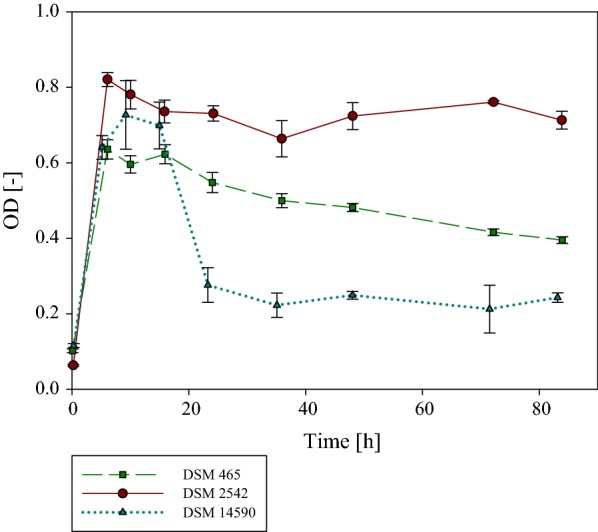
Growth curves of *P. thermoglucosidasius* DSM 2542^T^, *P. toebii* DSM 14590^T^ and *G. thermodenitrificans* DSM 465^T^. All strains were grown in quadruplicate in stoppered serum bottles with an initial gas atmosphere composition of 50% CO and 50% air. *P. thermoglucosidasius* DSM 2542^T^ reached a maximum absorbance (OD_600_ = 0.82) after 6.01 h, by the end of the cultivation the OD_600_ increased to a value of 0.71. A maximum absorbance for *P. toebii* DSM 14590^T^ was reached after 9.12 h (OD_600_ = 0.73). The OD_600_ decreased during the cultivation to a final value of 0.24. For *G. thermodenitrificans* DSM 465 the highest OD_600_ = 0.64 was observed after 6.04 h. The OD_600_ decreased to a final value of 0.40

The predicted function of the co-localized genes encoding the anaerobic CODH and H_2_-evolving hydrogenase (Fig. 3c) was tested using *P. thermoglucosidasius* DSM 2542^T^. Two related strains, *Geobacillus thermodenitrificans* DSM 465^T^ and *P. toebii* DSM 14590^T^, which lack both orthologues of the three hydrogenases and the anaerobic CODH, were included as controls. The cultivation of *P. thermoglucosidasius* DSM 2542^T^ in serum bottles with a gas atmosphere consisting of 50% CO and 50% air showed that this strain was able to effectively grow in the presence of 50% CO, reaching a maximum absorbance of 0.82 after 6 h of cultivation (Fig. 5). A fractional amount of CO was consumed at the beginning of the experiment, when oxygen was still available, by *P. toebii* DSM 14590^T^ (0.37 mmol) and *G. thermodenitrificans* DSM 465^T^ (0.216 mmol), respectively. This suggests that these strains may possess an alternative mechanism, such as an aerobic CO dehydrogenase, where CO oxidation is coupled to an electron transport chain which finally reduces oxygen [38]. For instance, a predicted aerobic CODH is present (CoxMSL—OXB91742-744) in *P. toebii* DSM 14590^T^ but is absent from *G. thermodenitrificans* DSM 465^T^.

**Fig. 6:**
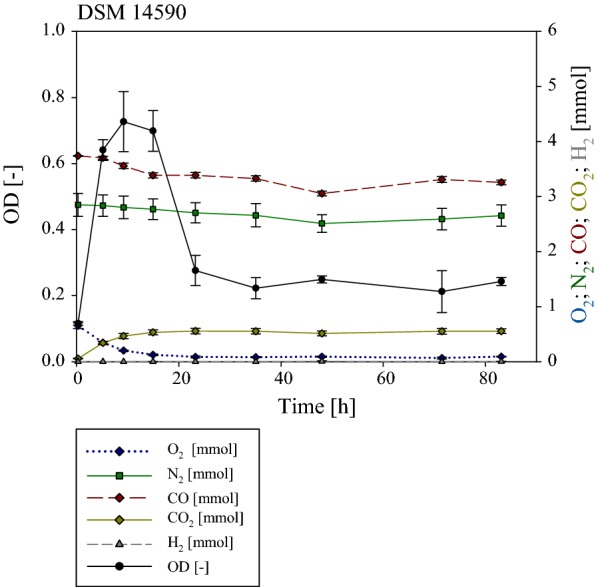
Gas phase composition during the cultivation of *P. toebii* DSM 14590^T^. Initial gas composition was 50% CO and 50% air. O_2_ (dark blue) decreased from 0.66 to ~ 0.01 mmol after 23.25 h. CO (dark red) decreased fractionally about 0.34 mmol. No hydrogen (dark grey) was detected. CO_2_ (dark yellow) increased during the cultivation to 0.56 mmol. After 9.12 h a maximum absorbance (OD_600_ in black) with a value of 0.73 was reached

**Fig. 7:**
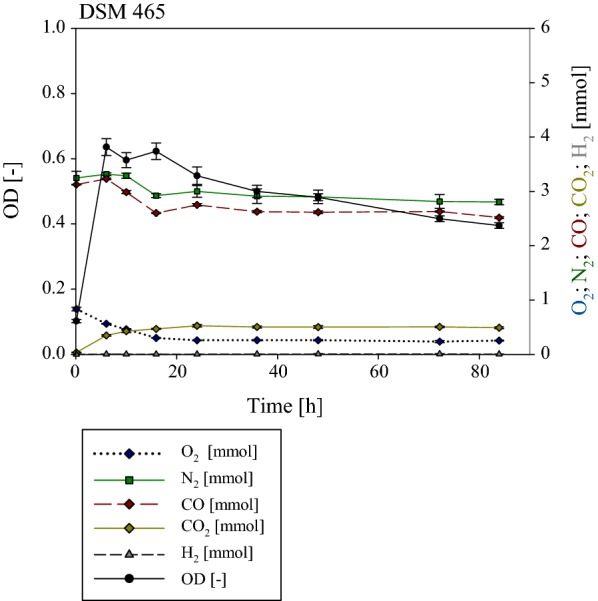
Gas phase composition during the cultivation of *G. thermodenitrificans* DSM 465. Initial gas composition was 50% CO and 50% air. O_2_ (dark blue) decreased from 0.83 to ~ 0.03 mmol after 24.01 h. CO (dark red) decreased fractionally about 0.22 mmol. No hydrogen (dark grey) was detected. CO_2_ (dark yellow) increased during the cultivation to 0.49 mmol. After 6.04 h a maximum absorbance (OD_600_ in black) with a value of 0.64 could be detected

**Fig. 8:**
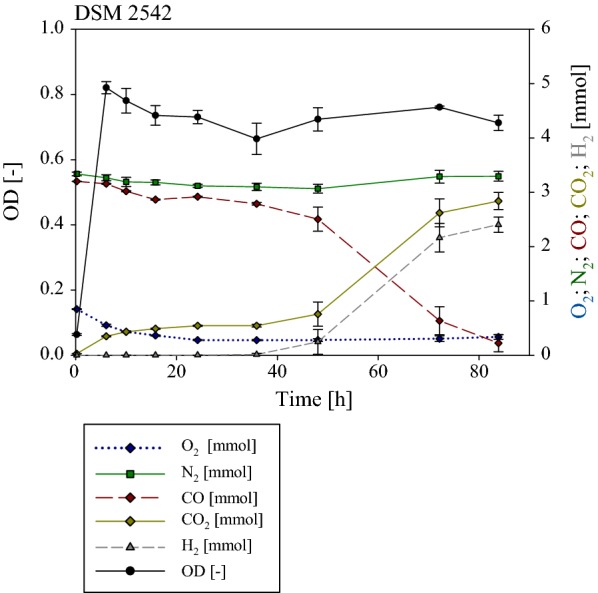
Gas phase composition during the cultivation of *P. thermoglucosidasius* DSM 2542^T^. Initial gas composition was 50% CO and 50% air. O_2_ (dark blue) decreased from 0.85 to ~ 0.03 mmol after 22 h. CO (dark red) decreased until the start of hydrogen production (dark grey) from 3.20 to 2.79 mmol (35.89 h). After 84 h the CO was consumed completely and 2.47 mmol hydrogen was produced. CO_2_ (dark yellow) increased during the cultivation to 2.84 mmol. After 6.01 h a maximum absorbance (OD_600_ in black) with a value of 0.82 was reached

While the two control strains tolerated the presence of CO, no H_2_ production was observed for either strain (Figs. 6 and 7). By contrast GC analyses revealed the production of H_2_ by *P. thermoglucosidasius* DSM 2542^T^ after ~ 36 h (Fig. 8). This corresponds with O_2_ reaching a plateau value of ~ 0.03 mmol. After 84 h 2.28 mmol CO was consumed and 2.47 mmol H_2_ produced. *P. thermoglucosidasius* DSM 2542^T^ is thus capable of producing H_2_ at a near equimolar conversion to CO consumption once most residual oxygen has been exhausted with a final yield of 1.08 H_2_/CO.

## Discussion

The redox potential and diffusion coefficient of molecular H_2_ make it a key component of metabolism and a potent energy source for many microbial taxa [25]. The ability to utilize this energy source relies on the production of various hydrogenase enzymes, which power both the consumption and production of H_2_ and inextricably couple H_2_ to energy-yielding pathways such as acetogenesis, methanogenesis and respiration [26, 43]. Our comparative genomic analysis revealed that *P. thermoglucosidasius* contains a unique hydrogenase compliment comprised of two uptake hydrogenases (group 1d and 2a) and one H_2_-evolving hydrogenase (group 4a). Evolutionary analysis showed that these hydrogenases are derived through three independent evolutionary events. This indicates that H_2_ is likely to play a pivotal role in *P. thermoglucosidasius* metabolism and bioenergetics in the ecological niches it occupies. By contrast, members of the sister genus *Geobacillus* lack orthologous hydrogenase loci and, aside from *P. thermoglucosidasius*, only the group 1d and 2a uptake hydrogenases share orthology in one and three *Parageobacillus* spp., respectively, even though they are frequently isolated from the same environments.

The group 4a H_2_-evolving hydrogenase of *P. thermoglucosidasius* is not found in any other members of the class Bacilli and is most closely related to those found in members belonging to the class Clostridia, particularly the family *Thermoanaerobacteraceae*. Furthermore, it forms an association with a CODH, which is found in common with a more restricted subclade of strict anaerobes within the family *Thermoanaerobacteraceae*. Our fermentation studies with *P. thermoglucosidasius* in the presence of CO showed that *P. thermoglucosidasius* grows efficiently when exposed to high concentrations of CO and that the CODH-group 4a hydrogenase complex can effectively couple CO oxidation to H_2_ evolution, *P. thermoglucosidasius* can do so at a near-equimolar conversion. Furthermore, unlike other CO oxidizing hydrogenogenic bacteria, which are strict anaerobes, *P. thermoglucosidasius* is a facultative anaerobe capable of first removing residual oxygen from CO gas sources prior to producing H_2_ via the water-gas shift reaction. The combination of these features makes *P. thermoglucosidasius* an attractive target for potential incorporation in industrial-scale production strategies of biohydrogen.

## Additional files

**Additional file 1.** Calculation of the gas composition. Description of the calculation of the gas composition by using the ideal gas law.

**Additional file 2.** Annotations of the CODH and [Ni-Fe] hydrogenase loci of *P. thermoglucosidasius* DSM 2542^T^. The locus tags, sizes, protein names as well as the functions of the proteins in the three [Ni-Fe] hydrogenase loci and the anaerobic CODH locus of *P. thermoglucosidasius* DSM 2542^T^. BlastP data (locus tag, average amino acid identity, bitscore and e-value) for the closest non-*Parageobacillus* orthologue and the top conserved domain for each *P. thermoglucosidasius* DSM 2542^T^ protein are shown.

**Additional file 3.** Orthologous [Ni-Fe] hydrogenase and anaerobic CODH loci in *Parageobacillus* and other taxa. The locus size, G+C content, G+C deviation of the orthologous [Ni-Fe] hydrogenase and anaerobic CODH loci of other *P. thermoglucosidasius* strains and distinct taxa as used in Figs. 2, 3 and 4. The number of protein orthologous and average amino acid identity of these proteins to those encoded on the *P. thermoglucosidasius* DSM 2542^T^ loci are indicated.
