# Supplementary material for: CO-dependent hydrogen production by the facultative anaerobe Parageobacillus thermoglucosidasius
Source: Microb Cell Fact. 2018 Jul 9;17:108. doi: 10.1186/s12934-018-0954-3 (PMC6036681; doi:10.1186/s12934-018-0954-3)
Supplement: Supplementary file 1 — Additional file 1. Calculation of the gas composition. Description of the calculation of the gas composition by using the ideal gas law. [file 12934_2018_954_MOESM1_ESM.docx]

**Additional file 1**

For calculation of the gas composition, the ideal gas law was used:

n = P * V / R * T (Eq. 1)

Where n = number of mols of gas; P = pressure of gas (1.013 bar + gas mixture over/under-pressure (*p*) * 10^5^); V = volume of the gas (Start V - number of ml removed prior to each GC measurement); R = universal gas constant (8.314 J * Kelvin^-1^ * mol^-1^ or kg * m^2^sec^-2^mol^-1^Kelvin^-1^_)_; T = growth temperature of cells (333.15 K (60 °C))

The formula for calculation of the gas amount was thus:

n_TOTAL_ = (1.013 + *p*) * 10^2^ * V / 2769.8091 (Eq. 3)

Due to the fact that water could be present in the gas phase at 60 °C, the number of moles of water must be subtracted from the number of moles of the total gas:

C_H2O_ = 130 mg * L^-3^

m_H2O_ = C * V (in mg)

n_H2O_ = m /Mr

n_GAS_ = n_TOTAL_ – n_H2O_

This was used for the final gas concentration formula of:

E.g. for H_2_: n_H2_ = n_GAS_ * GC % of H_2_ (or mol % of H_2_) /100 (Eq. 4)
